# Data to understand the biotransfer of heavy metals along the soil-plant-edible insect-human food chain in Africa

**DOI:** 10.1016/j.dib.2023.109434

**Published:** 2023-07-20

**Authors:** Susan Mwelwa, Donald Chungu, Frank Tailoka, Dennis Beesigamukama, Chrysantus M. Tanga

**Affiliations:** a Biology Department, School of Graduate Studies, Copperbelt University, P.O Box 21692, Kitwe, Zambia; b Cavendish University, Corner of Great North and Washama Roads, Villa Elizabeth, Zambia; c Mukuba University, P.O Box 20382, Kitwe, Zambia; d International Center for Insect Physiology and Ecology, P.O Box 30772-00100, Nairobi, Kenya

**Keywords:** Mining, Heavy metals, Edible insects, Contamination, Food safety

## Abstract

Data on the biotransfer of heavy metals along the soil-plant-edible insect-human food chain collected along a 60km pollution gradient is presented here. These datasets consists of concentrations of eight heavy metals (Arsenic, Cadmium, Copper, Chromium, Iron, Nickel, Lead and Zinc) in the soils, in five host plants species, and in seven edible insect species determined using Atomic Absorption Spectrophotometry (AAS). Datasets for the daily intake of metals and target hazard quotients for each edible insect species are also given. These data demonstrate the potential biotransfer of heavy metals along the soil-plant-edible insect-human food chain, and that edible insects harvested in heavy metal-polluted environments could pose serious health risks. These datasets provide further understanding of the relationships among metal concentrations in the soils, host plants and edible insects, particularly in the mining regions. For further details, refer to the article, “Biotransfer of heavy metals along the soil-plant-edible insect-human food chain in Africa” Mwelwa et al., [1].

**Specifications Table**

**Table:** 

Subject	Biological sciences (Systematics, Ecology and Behavior, and Entomology and insect science)
Specific subject area	Metal accumulation in edible insects harvested from polluted environments, and their associated health risks.
Type of data	Tables Graphs Figures
How the data were acquired	The data were collected during the 2021-2022 edible insect collection season in Zambia. We used sweep nets for capturing grasshoppers, handpicking for collecting caterpillars, termites and host plant samples, and soil samples were collected using a soil auger (spade). Atomic Absorption Spectrophotometry (AAS) was used to determine the concentrations of arsenic (As), cadmium (Cd), chromium (Cr), copper (Cu), iron (Fe), nickel (Ni), lead (Pb) and zinc (Zn) in soil, edible insect and host plant samples. The daily consumption of edible insect species was determined using a questionnaire. The concentrations of metals in edible insects and daily consumption were used to calculate the Estimated Daily Intake of Metals (EDIMs) and Target Hazard Quotients (THQs) as guided by US EPA. Statistical analyses were conducted using R version 4.1.0 while data summaries were generated in Microsoft excel.
Data format	Raw Analyzed Filtered
Description of data collection	Research assistants were trained to establish transects and sampling plots, administer questionnaires, and collect samples of soil, edible insects and host plants. Samples were collected along the 60 km heavy metal pollution gradient, with sampling sites at intervals of 15km. The first transect (sampling site) was established at 1.5km from the mine (nearest possible point). Along each transect, three 30 × 30m sampling plots (200m apart) were established.
Data source location	• City/Town/Region: Kitwe, Copperbelt province • Country: Zambia • Latitude and longitude (13°′ 01′ S, 27°′ 32′E.)
Data accessibility	Repository name: Mendeley Data. Data identification number: 10.17632/9bzvwnc2k3.1 Direct URL to data: https://data.mendeley.com/datasets/9bzvwnc2k3/1[8]
Related research article	S. Mwelwa, D. Chungu, F. Tailoka, D. Beesigamukama, C. M. Tanga, Biotransfer of heavy metals along the soil-plant-edible insect-human food chain in Africa. Sci. Total Environ. 881 (2023), p.163150. https://doi.org/10.1016/j.scitotenv.2023.163150

## Value of the Data

• The data provides insight into the biotransfer of heavy metals from the soil to host plants to edible insects to humans.
• These datasets can be a benchmark for future research on the impact of mining activities on the safety of wildly harvested edible insects.
• These data could inform policy on minimization of contamination of edible insects and wildlife conservation in polluted environments.
• Data on Estimated Daily Intake of Metals (EDIMs) and Target Hazard Quotients (THQs) associated with the consumption of edible insect species may provide the basis for quality control and regulation of entomophagy, and transformation from wild harvesting of edible insects to captive mass rearing to reduce chemical contamination.

## 1 Objective

These datasets are aimed at presenting data on biotransfer of heavy metals along the soil-plant-edible insect-human food chain in Africa. These datasets are valuable to the associated article Mwelwa et al. [1] in that raw, filtered and analyzed datasets on the kind and extent of the relationship among metal concentrations in soil, distance from the mine, host plants and edible insects are clearly presented. Furthermore, the estimated daily consumption (Dc) dataset for each edible insect species could inform practice in the emerging insects for food and feed industry, particularly in mass production.

## 2 Data Description

**Fig. 1:**
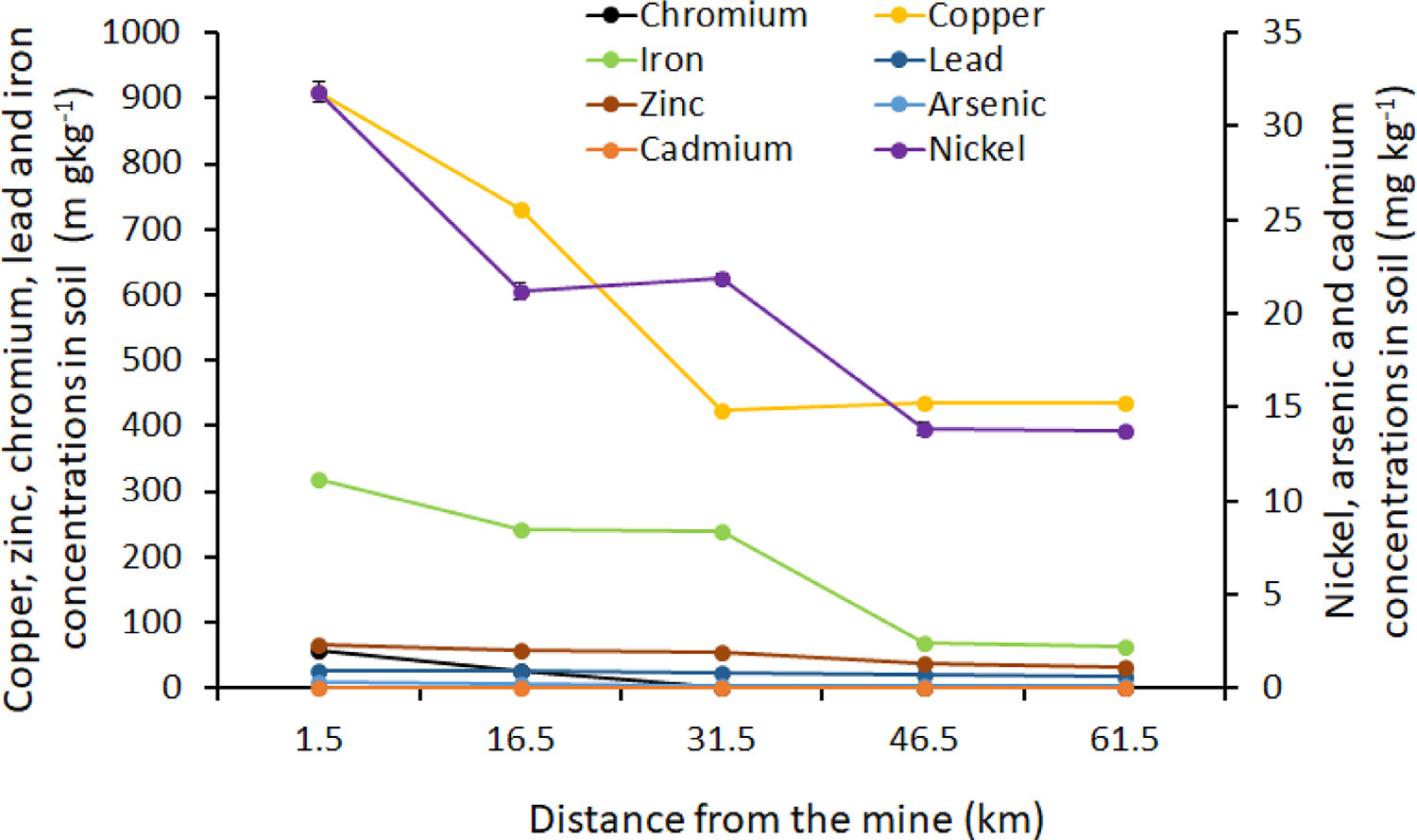
Metal concentrations in soils (mgkg^−1^) along the pollution gradient during the period 2021-2022 in the Copperbelt Province, Zambia.

**Fig. 2:**
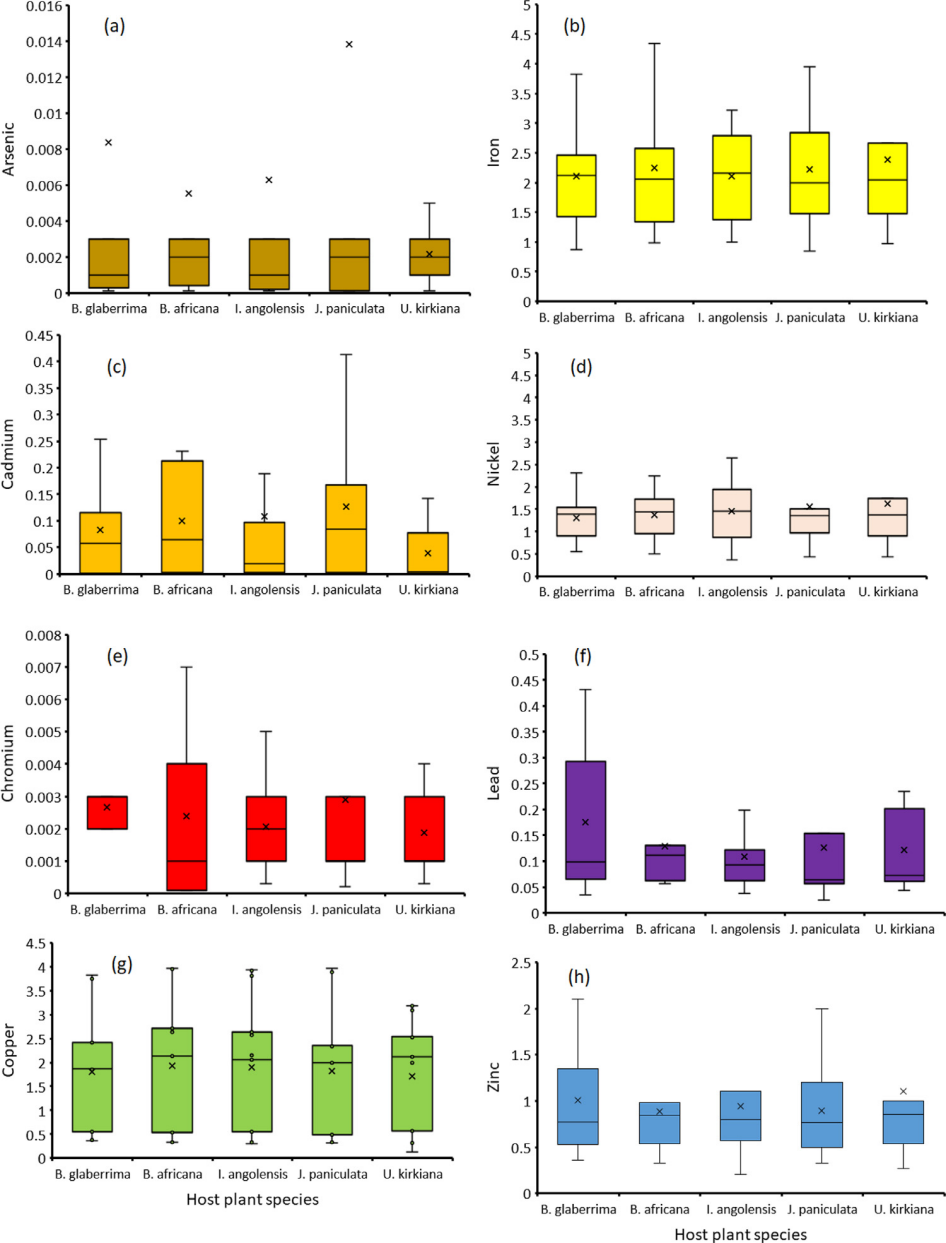
Metal concentrations in host plants (mgkg^−1^) along a 60 km pollution gradient during the period 2021-2022 in the Copperbelt Province, Zambia.

**Fig. 3:**
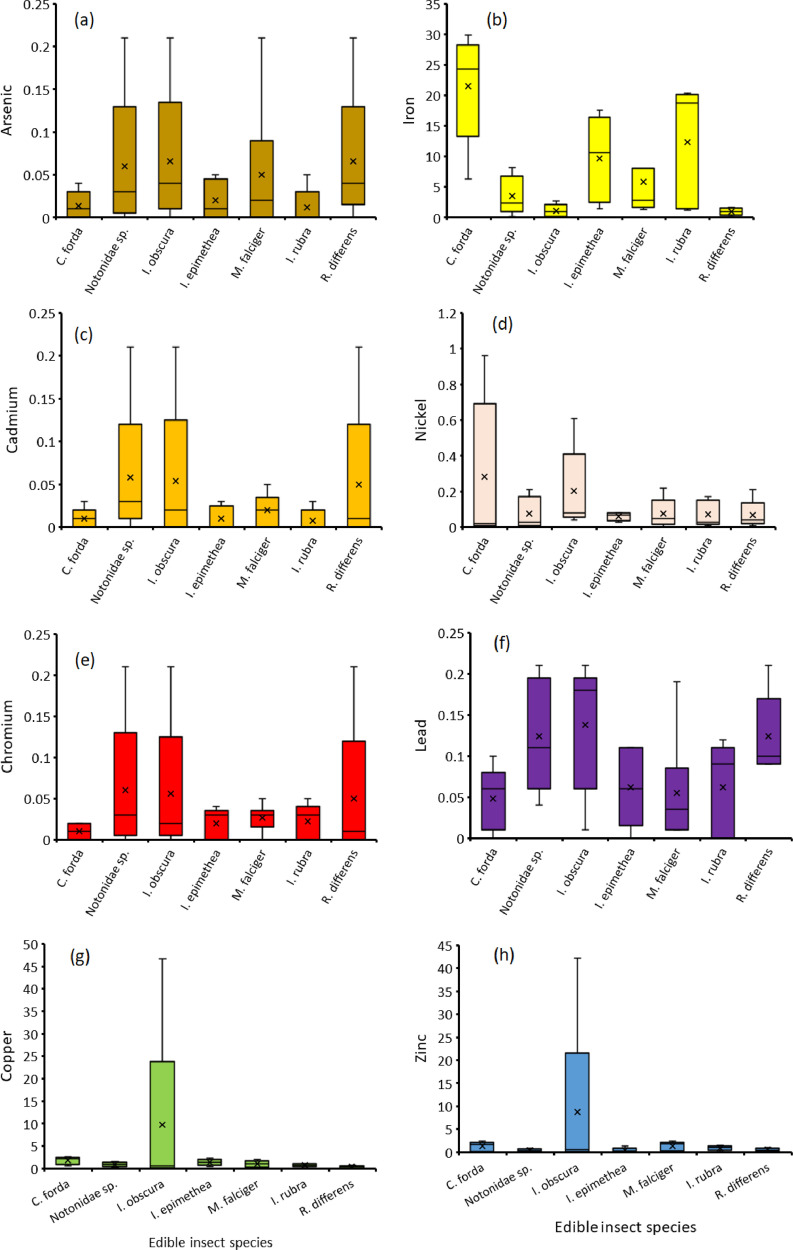
Metal concentrations in edible insects (mgkg^−1^) along a 60 km pollution gradient during the period 2021-2022 in the Copperbelt Province, Zambia.

**Fig. 4:**
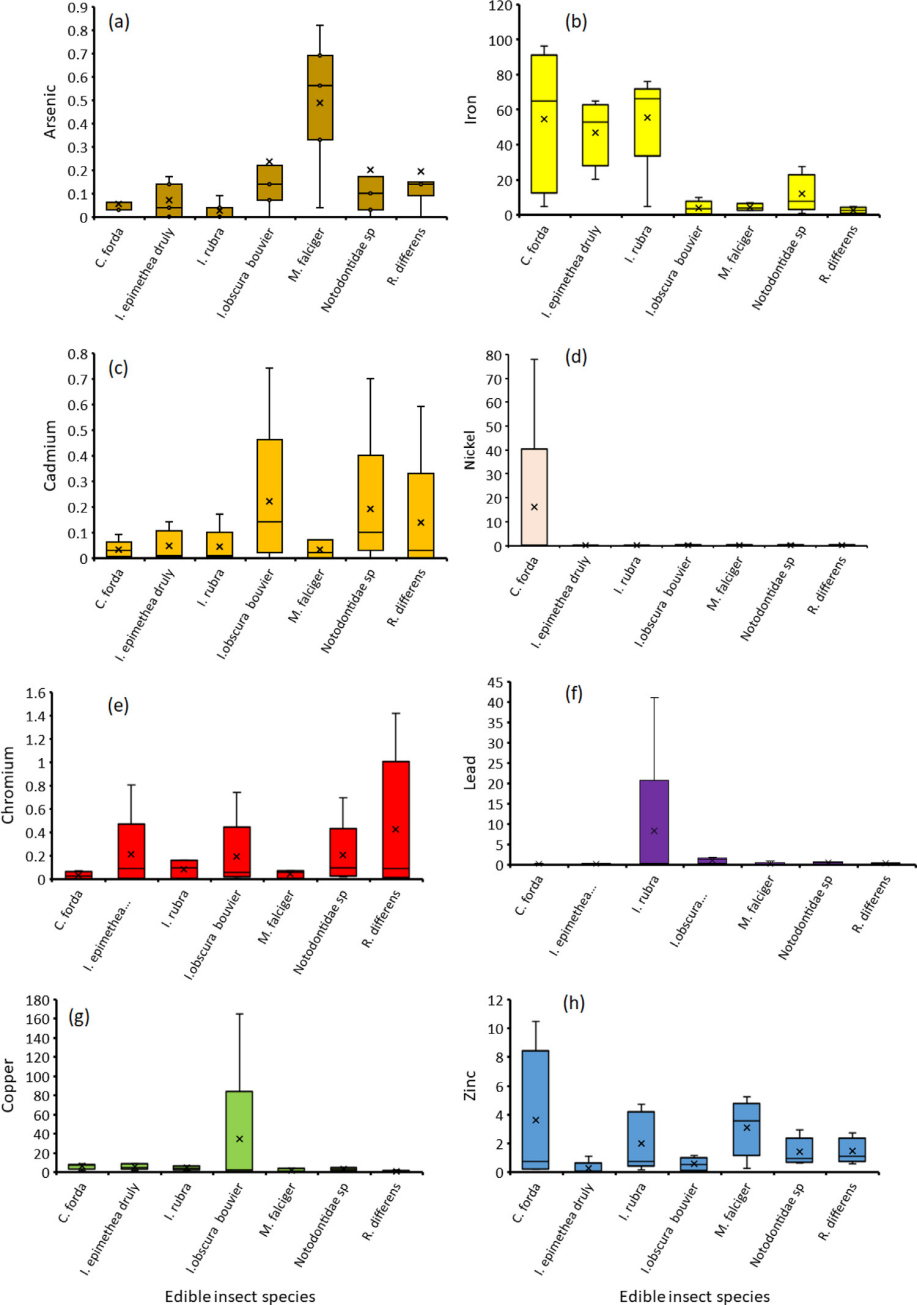
Estimated daily intake of metals (mgkg^−1^) resulting from edible insect consumption in the Copperbelt province, Zambia.

**Fig. 5:**
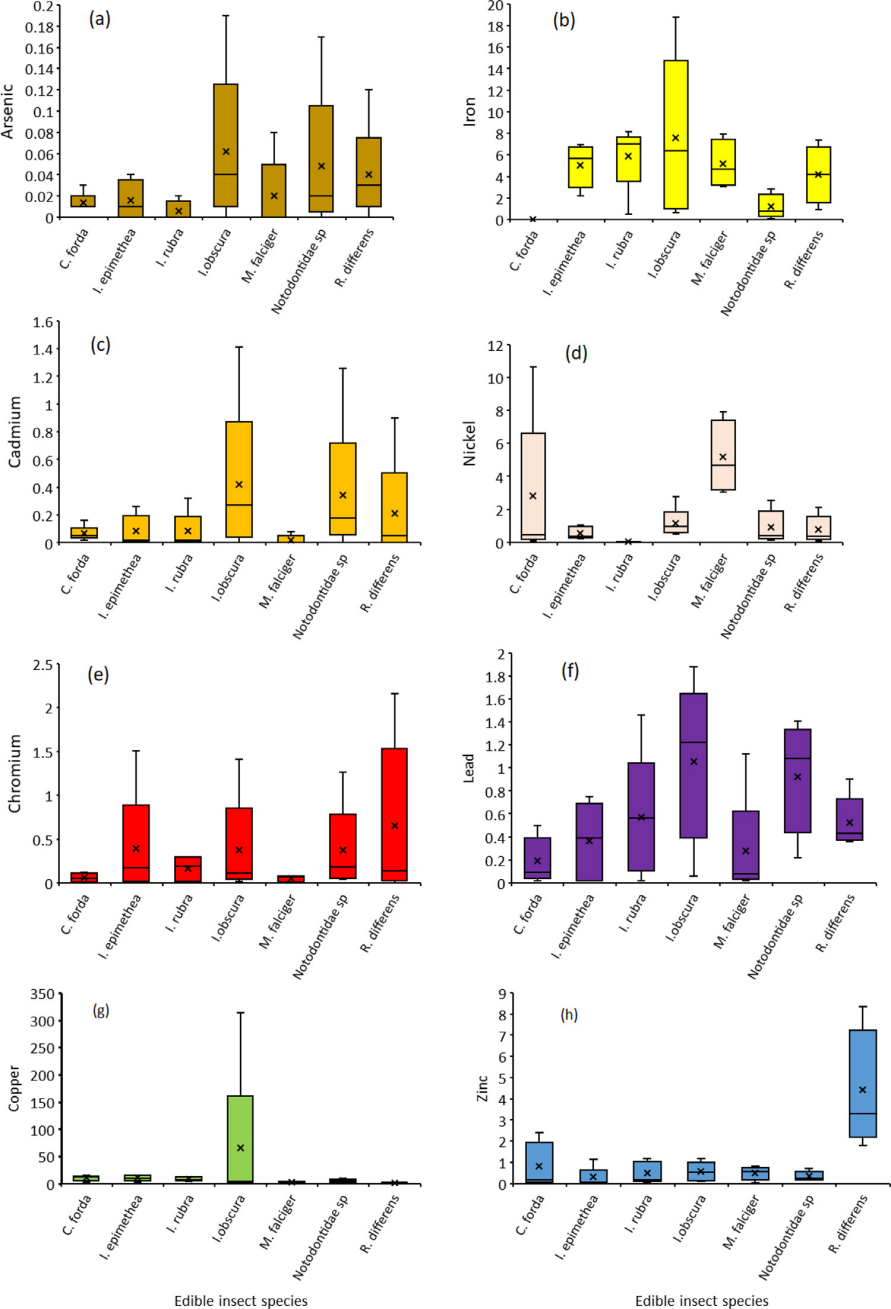
Target Hazard Quotients associated with edible insects in the Copperbelt province, Zambia.

**Fig. 6:**
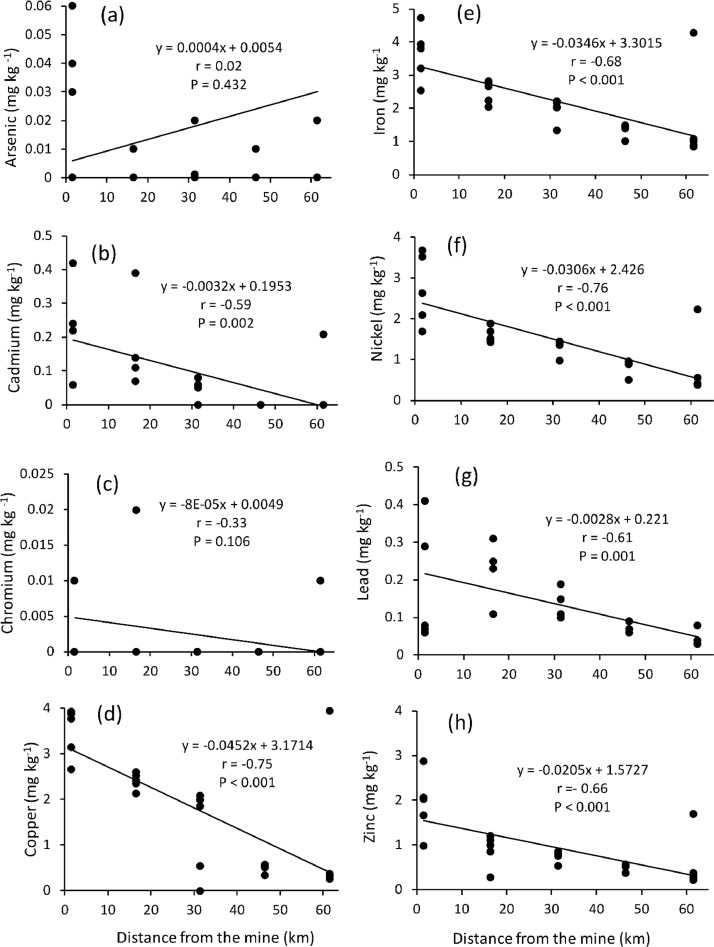
Relationship between concentrations of metals in the soil and distance from the mine.

**Fig. 7:**
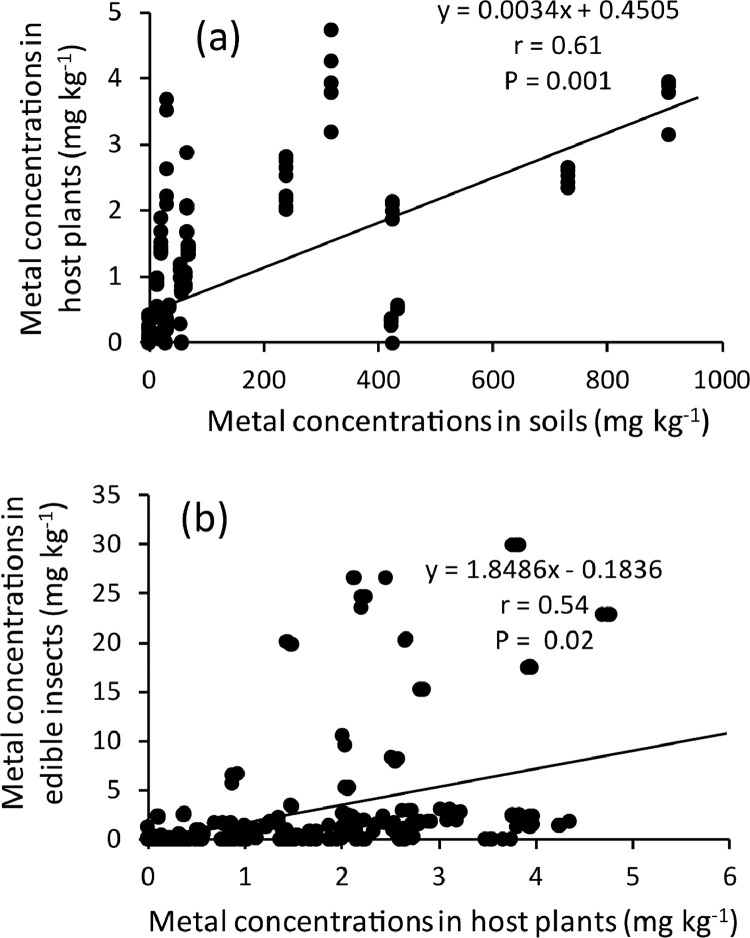
Relationship between metal concentrations in the soils and host plants.

The orders Isoptera, Lepidoptera and Orthoptera comprises some of the most commonly consumed insects in Africa. On the other hand, As, Cd, Cu, Cr, Fe, Ni, Pb and Zn are the common heavy metals found in elevated levels in the mining regions of Africa, including the Coppernelt province of Zambia. The datasets presented here, therefore, include metal concentrations in the soil, five host plants and seven edible insects from the earlier mentioned orders along the 60km stretch. EDI via consumption of different edible insect species and the THQs for each of the metals investigated. These datasets are summarized in Figs. 1, 2, 3, Fig. 4, Fig. 5. The raw data for the concentrations of As, Cd, Cu, Cr, Fe, Ni, Pb and Zn in the soils and host plants with distance from the mines, as well as the concentrations of these metals in *C. forda, I. obscura, I. rubra, I. epimethea, M. falciger* and *R. differens* in the Copperbelt Province are presented in the Mendeley repository. In addition, data for host plant tissue metal concentrations were pooled and correlated with distance from the mine. Similarly, host plant tissue metal concentrations were correlated with the edible insect tissue metal concentrations while soil metal concentrations were correlated with distance from the mine to establish relationships along a pollution gradient. Furthermore, data on the estimated daily intake of metals associated with the consumption of the above-mentioned insects was determined using a simple questionnaire. The local people where asked several questions including how much of each insect they consume per day and for how long in a year, the questionnaire also had a provision for allowing the respondents to put the insects they felt a person consumes per day in a bow that was provided to them. These insects were then weighed, and the quantity recorded. The questionnaire was uploaded in the Mendeley repository and can be accessed through the link provided in the data accessibility section of this paper. The datasets for metal concentrations in soils, host plants and edible insects are presented as correlation plots in Fig. 6, Fig. 7.

## 3 Experimental Design, Materials and Methods

### 3.1 Collection of edible insects, host plants, and soil samples

Edible insects, host plants and soil samples were collected along a 60km heavy metal pollution gradient. The first sampling site was established 1.5km from the mine. The second, third, fourth and fifth sites were established at 16.5km, 31.5km, 46.5km and 61.5km from the mine respectively. At each sampling site, an 800m transect perpendicular to the contamination gradient (wind direction) was established, and along this transect, three plots of 30 × 30m were established at intervals of 200m. From each study plot 75 soil samples (0-10cm depth), 200 host plant samples and 280 edible insect samples were collected. Plant and insect (caterpillar and termite) samples were collected by handpicking while sweep nets were used to capture flying insects (grasshoppers). The edibility status of the insect samples was determined on-site with the help of the local people Chungu et al. [2]. All the samples were kept in plastic vials, labelled appropriately and transported to the Copperbelt University laboratory for processing and analysis Azam et al. [3]. The insects and host plant samples were identified by entomologists and taxonomists at the Copperbelt University in Kitwe using identification keys for the region Séré et al. [4]. In the laboratory, insects and plant samples were cleaned of debris using deionized water, air-dried for seven days, and oven-dried for 24 hours at 105 °C. After oven drying, insect and plant samples were separately ground into powder using a motor and pestle pending heavy metal analysis. Soil samples were air-dried for seven days, ground, and sieved through a 2mm mesh sieve, pending laboratory analysis.

### 3.2 Determination of heavy metal concentration in soils, edible insects, and host plants

The concentrations of Cu, Zn, Ni, Fe, and Pb were determined using atomic absorption spectrophotometer (AAS; model Analyst 200, PerkinElmer Inc., Shelton, USA) while Cd, As, and Cr concentrations were determined using ICP-OES Turhan et al. [5]. Following the manufacturers guidelines, 1 g each of insect and plant samples was separately digested in 25ml of concentrated nitric acid at 250 °C for 50 minutes. This was repeated with 10ml of Per chloric acid. After cooling, 30 ml of deionized water was added, and the solution was reheated for 30 minutes. The solution was allowed to cool and then diluted to 50ml using deionized water pending reading on AAS. Regarding soil samples, 2g of each soil sample was put in beakers, and then 3-4 drops of Hydrofluoric acid (H.F) were added to each sample. After that, 30ml of Nitric acid was added to each sample and then heated on the hot plate for 30 minutes. After cooling, the samples were diluted up to 100ml with distilled water using 100ml conical flasks. The samples in the flasks were then thoroughly shaken and later filtered using filter papers. The filtrate was diluted up to 50ml and aspirated on an AAS to determine their metal concentrations Turhan et al. [5]. The wavelengths for the lamps used were 324.75, 283.31,213.86, 248.33, and 232.00 for Cu, Pb, Zn, Fe, and Ni, respectively Wasim et al., [6].

### 3.3 Calculation of EDIMs

The estimated daily intake (EDI) of each heavy metal was calculated as a product of daily insect consumption (Dc) (gd^−1^) and mean metal concentration in each insect species (mgkg^−1^) and weighted by average adult body weight W (kg) Eq. (1).

(1) $$\text{EDI}=\frac{D_{C}\;\times \;C_{m}}{W}$$

Dc was estimated by interviewing local people practicing entomophagy in the study site (250 adults: 56 males and 194 females) using a questionnaire. W was taken to be 70.3 kg, the average weight for adults in Southern Africa, where Zambia is located.

### 3.4 Estimation of target hazard quotient

The risk associated with the intake of a particular metal (THQ) was taken as the proportion of how long a certain amount of metal is consumed in a year and the recommended daily consumption limit of the heavy metal Eq. (2).

(2) $$\text{THQ}=0.001\frac{E_{f}\;\times \;E_{d}\;\times \;D_{c}\;\times \;C_{m}}{\text{RfD}\;\times \;W\;\times \;T}\;$$

Where,

THQ is the health hazard caused by a single metal, E_f_ is duration of exposure in a year, E_d_ the average time (years) respondents have stayed in the study area until interview time, D_c_ is the quantity of insects consumed per day; C_m_ is the quantified amount of metals in insects; RfD is the recommended limits (mg kg^−1^ d^−1^); T is the mean exposure duration for non-carcinogens (365 days’ year^−1^ × E_d_) US EPA, [7].

In view of the fact that the health risk is usually as a result of exposure to several metals, the risk posed by all the metals [hazard index (HI)] quantified in the insects investigated in this study was established using Eq. (3).

(3) HI=∑THQ

Where HI is the risk posed by more than one metal; THQ is the target hazard quotient.

## Ethics Statement

The research ethics review committee of Copperbelt University reviewed the research design and data collection tools and approved the study. Oral consent was sought from the respondents during the data collection, after being provided with sufficient information about the research to allow them to make informed and independent decisions regarding their participation in the survey.

## CRediT Author Statement

**Susan Mwelwa:** Conceptualization, Methodology, Investigation, Formal Analysis, Data Curation, Writing – original Draft; **Donald Chungu:** Supervision, Project Administration, Software, Writing – review & editing, Validation; **Frank Tailoka:** Supervision, Project Administration, Software, Writing – review & editing, Validation; **Dennis Beesigamukama:** Formal Analysis, Data curation, Writing – review & editing, Validation; **Chrysantus M. Tanga:** Supervision, Funding, Resources, Project Administration, Software, Writing – review & editing, Validation.

## Data Availability

Biotransfer data (Original data) (Mendeley Data).
